# Which factors have an association to the Quality of Life (QoL) of people with acquired Spinal Cord Injury (SCI)? A cross-sectional explorative observational study

**DOI:** 10.1038/s41393-021-00663-z

**Published:** 2021-07-08

**Authors:** Christian Sturm, Christoph M. Gutenbrunner, Christoph Egen, Veronika Geng, Christina Lemhöfer, Yorck B. Kalke, Christoph Korallus, Roland Thietje, Thomas Liebscher, Rainer Abel, Andrea Bökel

**Affiliations:** 1 Department of Rehabilitation Medicine, Hannover Medical School, Hanover, Germany; 2 Manfred-Sauer-Foundation, Lobbach, Germany; 3 Institute for Physiotherapy, University Hospital Jena, Jena, Germany; 4 RKU – University and Rehabilitation Clinics Ulm, Ulm, Germany; 5 Center for spinal injuries, Trauma Hospital Hamburg, Hamburg, Germany; 6 Treatment Centre for Spinal Cord Injuries, Trauma Hospital Berlin, Berlin, Germany; 7 SCI Unit, Klinikum Bayreuth GmbH, Bayreuth, Germany

**Keywords:** Outcomes research, Population screening

## Abstract

**Study design:**

Cross-sectional explorative observational study.

**Objectives:**

To identify factors which have an association to the self-perceived Quality of Life (QoL) for persons with acquired spinal cord injury (SCI).

**Setting:**

Eight specialized SCI-centers in Germany. The GerSCI survey is the German part of the International Spinal Cord Injury Survey (InSCI).

**Methods:**

Self-disclosure questionnaire, created from the InSCI group, translated and adapted for Germany. The questionnaire collects a very broad range of data and, and due to its design as a self-report, is particularly suitable for the analysis on QoL. Because of the content, which is binding for all participating states, it allows a direct comparability of the results. Included in Germany were 1479 persons with acquired SCI aged 18 years and older.

**Results:**

Various factors were identified with high associations to QoL, including changeable and unchangeable ones, such as those of particular importance: pain, sleep problems, sexual dysfunction, age, and time since onset of SCI. Some results confirmed reports of previous studies, others were surprising.

**Conclusion:**

this study provides an important basis for the planned analysis of the InSCI participating countries in the 6 WHO regions. Germany was able to contribute the largest study population. The concrete study design of InSCI allows us to directly compare data and helps us to improve ourselves within the framework of a “learning health system”. Medical measures can be orientated towards the found results, in order to ensure the best possible care and support by the therapeutic team, individually adapted to the person, place of residence and impairment.

## Introduction

### Current knowledge about Quality of Life with SCI

The life expectancy of patients after SCI has increased significantly almost everywhere in the world due to better acute medical care, although there are still large international differences [1]. For this very reason, a good acute and rehabilitative care including life-long treatment under consideration of the quality of life are becoming increasingly important.

While economic evaluations typically embrace health maximization as the maximization objective, using quality-adjusted life years, there is increasing interest in measuring capability well-being and subjective well-being for informing policy decision-makers [2].

In recent years, there have been a large amount of studies that have looked at, among other things, the impact on the quality of life of people with SCI. Only a few of the more newer ones can be named here as examples that attempt to reflect the state of knowledge. Factors that seem to have a particularly high association are pain and spasticity [3–6], as well as bladder and bowel function [7–9]. Sexual dysfunction also seems to have an association to QoL [6, 10, 11].

Studys and already meta-analysis showed the high impact to QoL of the psychological aspects. Greater acceptance of SCI, life satisfaction, level of depression and anxiety have associations to QoL [6, 12–16]. A great influence on the mental status seems to have the participation in social life [6, 16].

Healthy SCI individuals tend to have better QoL measures and secondary health issues after SCI are affecting QoL and social participation [17]. Older studies, but also very recent ones, confirm that the quality of life of adults with chronic SCI was lower compared with reference populations [6, 18].

In the past, it was already regretted that there was no single definition of Quality of Life that everyone agreed upon, largely due to the breadth of literature that addressed this topic and the varying definitions used in studies [19]. This, too, makes it difficult to compare results between populations or different states. International research projects such as InSCI could help to ensure that there is a uniform definition at some time.

### International background

Both the United Nations (UN) and the World Health Organization (WHO) demand the collection of internationally comparable data on the living and care situation of people with disabilities [20]. Consequently, the International Spinal Cord Injury Survey (InSCI) was launched, headed by the International Spinal Cord Society (ISCoS) and the International Society of Physical and Rehabilitation Medicine (ISPRM), within the framework of the WHO Collaboration Plan and the coordination of Swiss Paraplegic Research (SPF). The aim of the InSCI is for health systems to learn from each other through the comparison of results of the 22 participating countries in all 6 WHO regions. The outcomes should also help to develop recommendations for decision-makers in politics and health care [21]. Furthermore, reliable national data are also required to ensure optimal care and supply [22]. Germany is one of the participating countries to collect these requested data to compare within InSCI. In Germany the project is called “German Spinal Cord Injury Survey (GerSCI)”.

One special advantage of the InSCI survey is, that the persons concerned are asked about their point of view and their perceived situation. Therefore, this data collection is particularly suitable for the analysis of the factors potentially influencing perceived Quality of Life (QoL).

### Special aspects of this study

One of the first results of the GerSCI study was that, for persons with SCI, QoL decreased with increasing experience of barriers. Some of these aspects have already been highlighted by our study group [23], but it became clear that for this important topic of QoL a specific analysis of the different probably influencing factors was necessary. Therefore, we wanted to look at the different factors individually and try to put them into a clinical context.

In literature, a number of comprehensive questionnaires to assess QoL are available. They are based on diverse constructs of QoL and focus mainly on health-related performance in daily life. Another approach refers to a more general feeling of the subjective perception of life quality. Of course, subjective perception has multiple dimensions but also is related to the individual’s values and life goals. For pragmatic reasons and because the use of complex and extended questionnaires in our study would have led to be not user-friendly we decided to use a commonly used single question to get a rough estimate of subjective level of QoL. The evaluation of separate components and the differentiation between various constructs of functioning should be the subject of future studies.The specific aims of this study were: (i) to describe the level of Quality of Life in the German study population and (ii) to identify and describe the probability of influence on covariates of Quality of Life. (iii) to provide a basis for international comparison within InSCI.

### Methods

The German Spinal Cord Injury Survey (GerSCI) data set, as the German part of InSCI, served as the basis for the study. The used questionnaire was developed centrally by the InSCI study group and is thus binding for all InSCI participating countries. It has been shown, that the successful implementation of the InSCI survey enables the comparison of the situation of individuals with SCI in different regions around the world and constitutes a crucial starting point for an international learning experience [24]. GerSCI was implemented in 2017 and was conducted by the Department of Rehabilitation Medicine at Hannover Medical School.

### Inclusion criteria:

- Presence of acquired SCI (traumatic or non-traumatic)
- Age ≥ 18 years
- Completed post-acute rehabilitation: 12 months after the onset of the spinal cord lesion
- Current place of residence: Germany, language competence: German

### Exclusion criteria:

- Congenital SCI or neurodegenerative diseases

From the database of the eight participating specialized SCI centers, 5,598 potential

participants were identified. They were treated at least once in one of these specialized SCI clinics inpatient or outpatient. They received an invitation letter and the questionnaire, which they could answer in paper form or electronically. After the exclusion of questionnaires that failed to satisfy the inclusion criteria, the available participant data declined by *n* = 79. Some were excluded due to aborted online questionnaires (*n* = 56), received duplicate questionnaires (*n* = 2) and > 30 % of missing values (*n* = 138). 1479 questionnaires were considered for data evaluation [25].

The potential influence of various factors on QoL were studied using measurements of association.

### Survey instruments

#### Measuring quality of life (WHOQoL-BREF)

The WHOQoL-BREF is an instrument for recording subjective QoL. It is based on the definition of QoL, as the perception of one’s own life situation in the context of respective culture and value systems, as well as in relation to individual goals, expectations, and interests. We use the term QoL to refer to the perceived, purely subjective experience of the participants, in order to reflect their individual perspective in this study. We relate this statement to perceived barriers and enabling factors. The WHOQoL-BREF questionnaire consists of 26 items that focus on several dimensions, such as physical well-being, psychological well-being, social relationships, and environment [26]. Six items were used from the WHOQoL-BREF in the GerSCI questionnaire. These items were predetermined by the InSCI study group. Since there is no reliable sum score of this question selection given by the InSCI team, we chose the overall QoL assessment as the primary target criterion. However, regarding this study, the item which was used for associations as the main parameter was, “How would you rate your quality of life in the last 30 days?”, which were rated on a scale with five ratings from “very poor” to “very good”. Since not the complete WHOQoL-Bref was used in this questionnaire, we were able to create the WHOQoL-Bref global domain score with the first and second question using a scale transformation (0–100). This scale transformed global domain values were then compared to the general population in the year 2000 according to Angermeyer et al. [27]. Unfortunately, no matched and more recent comparison sample is available from Germany.

### Covariates

The selection of the covariates within the framework of the InSCI questionnaire was made based on the literature reports described in the introduction and expert discussion within the GerSCI team about possible additional relevant factors. In order to analyze for associations, sociodemographic data and lesion characteristics were used, including age, gender, relationship status, level of injury (paraplegia vs tetraplegia), injury severity (complete vs incomplete), time since injury, etiology (traumatic vs non-traumatic), satisfaction with community health services (satisfied vs not satisfied), difficulties gaining medical aids (no vs yes), employment status (unemployed vs employed), education level (low vs high), net household income (under average vs over average) and health conditions (no vs yes: sleep problems, bowel dysfunction, sexual dysfunction, contractures, decubitus, urinary bladder function, bladder infection, spasticity, respiratory problems, injuries due to sensory disorders, circulatory problems or circulatory disorders, dysreflexia, orthostatic hypotension, diabetes mellitus, periarticular ossification), and pain (no to mild, and moderate to severe).

The five rating categories of the WHOQoL-Bref were dichotomized into “0”, which corresponded with “very bad”, “bad” and “mediocre” and “1”, which corresponded with “well” and “very good”. The pain-scale with rating categories 0 to 10 was convertes in a dummy variable according to Ledowski et al. in 0–3 = *no to mild pain* and 4–10 = *moderate to severe pain* [28]. Education status was converted in a dummy variable as 1 = no school leaving certificate, primary school certificate, lower secondary graduation, secondary school graduation, and 2 = advanced technical college entrance qualification, Abitur (general university entrance qualification). Satisfaction with community health service was converted in a dummy variable as 1 = “very satisfied”, “satisfied” and 2 = “neither” or, “unsatisfied”, “very unsatisfied”. Net household income was categorized into 1 = “less than 981 €, 982–1345 €, 1346–1660 €, 1661–1990 €, 1991–2339 €, 2340–2732 €, 2337–3195 €”, and 2 = “3196–3819 €, 3820–4837 €, more than 4838 €” according to average monthly net income per private household in Germany [29]. Problematic health conditions during the last three months were rated on a 5 point Likert scale from “not problematic” until “extremely problematic”. In this analysis, we dichotomized the scale in 0 = “no problem” and 1 = “a little problematic” till “extremely problematic”.

### Statistical analysis

Sociodemographic data and SCI characteristics were presented as percentages or means with standard deviation (SD). The key focus of this study was the QoL of people with SCI in Germany. WHOQoL-Bref frequencies were presented as percentages. Only the data from participants with less than 30% missing values were included in the statistical analysis.

To analyze associations between QoL covariates, associations using Eta and Cramers’V were calculated.

The determinants of QoL covariates associated with the perception of a high quality of life were assessed using multivariable logistic regression and estimated odds ratios (OR) with a 95% Confidence interval (CI). Variables were included if they showed significant associations to QoL, which were all variables except marital status.

The proportion of missing values was less than 5%, therefore no imputation of missing values has been performed.

Results were considered statistically significant if *p* values were less than 0.05. Statistical analyses were performed with SPSS IBM 26.0.

## Results

The average age of the respondents was 55.3 years (SD: 14.6). The range was from 19 to 90 years. 51,2 %; stated a paraplegia; 48,8 % a tetraplegia. Further details of socio-demographic and lesion characteristics of study participants are available elsewhere [23].

**Fig. 1:**
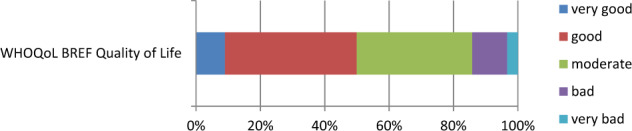
Comparison of the QoL of the study population compared to the norm sample. Response frequencies to the question: “How would you rate your quality of life in the last 30 days?”.

Most respondents answered the question “How would you rate your quality of life in the last 30 days?” with “good” (41%) to “moderate” (36%). A smaller proportion described it as “bad” (11%) or “very bad” (3.3%). A “very good” quality of life was reported by 9.0% (Fig. 1).

**Fig. 2:**
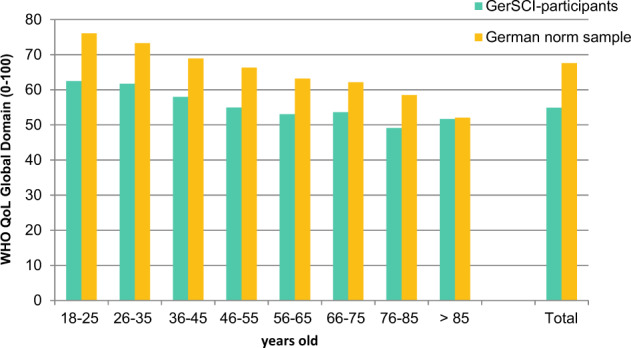
Results of factors associated with a high QoL. Comparison of the QoL of the study population with the norm sample description.

Fig. 2 shows the comparison of the QoL of the study population in relation to the available norm sample [27]. With regard to the age groups, it can be seen that the values of the norm sample are higher than the values of the study population in all age groups. In the age group > 85 years they are almost the same.

For almost all values selected from clinical experience and literature research, the association analysis showed a high significance with the development of QoL of *p* < 0.05, and most of them were even *p* < 0.001 (Tables 1 and 2).

**Table 1:** Results of contingency analysis with nominal data using Cramers’V.

Independent variable correlated with QoL	*n*	*p*	Cramers‘ V
Gender	1409	**0.001**	0.085
Lesion height	1376	**0.027**	0.060
Completeness of lesion	1402	**0.008**	0.070
SCI cause	1397	**<0.001**	0.131
Education level	1405	**0.010**	0.069
Net household income	1282	**<0.001**	0.209
Relationship status	1428	0.262	0.030
Employed in working age	1054	**<0.001**	0.260
Satisfaction community health services	1405	**<0.001**	0.140
Difficulties gaining medical aids	1388	**<0.001**	0.124
Sleep problems	1406	**<0.001**	0.306
Bowel dysfunction	1401	**<0.001**	0.289
Urinary tract infections	1389	**<0.001**	0.146
Urinary bladder dysfunction	1389	**<0.001**	0.203
Sexual dysfunction	1304	**<0.001**	0.306
Contractures	1376	**<0.001**	0.258
Spasticity	1411	**<0.001**	0.153
Decubitus	1396	**<0.001**	0.165
Respiratory problems	1388	**<0.001**	0.219
Injuries due to sensory disorders	1388	**<0.001**	0.154
Circulatory problems or circulatory disorders	1403	**<0.001**	0.203
Dysreflexia	1400	**<0.001**	0.230
Orthostatic hypotension	1386	**<0.001**	0.220
Pain	1404	**<0.001**	0.358
Joint and muscle pain	1409	**<0.001**	0.297
Diabetes mellitus	1386	**<0.001**	0.148
Periarticular ossification	1339	**<0.001**	0.129

*P* values < 0.05 are marked in bold.

**Table 2:** Results of contingency analysis with metric data using Eta.

Independent variable correlated with QoL	*n*	*p*	Eta
Age	1426	**<0.001**	0.168
Time since injury	1402	**0.007**	0.118

*P* values < 0.05 are marked in bold.

Due to this high levels of significance of most of the chosen factors, it is important to consider the effect size in order to assess the relevance of the factors. The largest effect size with Cramers’ V above 0.30 (Interpretation of Cramér’s V according to Cohen: small = 0.10; medium = 0.30; large = 0.50) were for pain, sleep problems, and sexual dysfunction. Even this does not say anything statistically accurate about the absolute level of association.

A logistic regression analysis shows that both the model as a whole (chi-square(27) = 249.695, *p* < 0.001) and some of the individual coefficients of the variables are significant. The R-square after Nagelkerke is 0.403, which explains 40.3% of the regression model.

The results of the logistic regression are displayed in Table 3. A higher relative probability of experiencing a good QoL is linked to people who are satisfied with their community health services (OR = 1.730; *p* = 0.013), those who have no difficulties in gaining medical aids (OR = 2.176; *p* < 0.001), who have no bowel dysfunction (OR = 1.774; *p* = 0.023), no sexual dysfunction (OR = 1.969; *p* = 0.027), no contractures (OR = 1.626; *p* = 0.032), no to mild pain (OR = 3.289; *p* < 0.001) and no diabetes mellitus (OR = 2.013; *p* = 0.039). Those who are unemployed (OR = 0.562; *p* = 0.004) have a higher relative probability of not experiencing a high QoL compared to employed participants. Converted from the data it can be concluded that with every year of life, the relative probability of a good QoL decreases by 3.1% and increases by 2.4% with each additional year of occurrence of SCI.

**Table 3:** Logistic regression for factors associated with the perception of a high QoL.

Variables				
Chi^2^(27)=249,695, *p* < 0.001			95% confidence interval	
Nagelkerkes *R*^2^ = 0.403	*p*	OR	Lower value	Upper value
Age	001	969	951	987
Gender (male vs female)	192	1.342	862	2.088
Para vs tetra	787	946	629	1.420
Complete vs incomplete	647	904	586	1.394
Time since injury	013	1.024	1.005	1.043
Aetiology (traumatic vs non-traumatic)	237	1.336	827	2.160
Satisfaction community health services (satisfied vs not satisfied)	013	1.730	1.124	2.663
Difficulties gaining medical aids (no vs yes)	000	2.167	1.461	3.216
Employment status (unemployed vs employed)	004	562	0.380	830
Educational level (low vs high)	675	918	615	1.370
Net household income (under average vs over average)	070	656	416	1.036
Sleep problems (no vs yes)	394	1.215	776	1.903
Bowel dysfunction (no vs yes)	023	1.774	1.083	2.907
Sexual dysfunction (no vs yes)	027	1.969	1.081	3.588
Contractures (no vs yes)	032	1.626	1.042	2.539
Decubitus (no vs yes)	101	1.425	0.934	2.175
Pain (no/mild vs moderate/severe pain)	000	3.289	2.200	4.919
Urinary bladder dysfunction (no vs yes)	939	1.016	0.669	1.544
Bladder infection (no vs yes)	376	0.820	529	1.272
Spasticity (no vs yes)	767	1.076	662	1.749
Respiratory problems (no vs yes)	389	1.207	787	1.852
Injuries due to sensory disorders (no vs yes)	703	1.087	708	1.668
Circulatory problems or circulatory disorders (no vs yes)	154	721	460	1.131
Dysreflexia (no vs yes)	214	1.304	858	1.983
Orthostatic hypotension (no vs yes)	187	1.334	870	2.047
Diabetes mellitus (no vs yes)	039	2.013	1.038	3.904
Periarticular ossification (no vs yes)	374	1.271	749	2.158

The reference category was the last one, e.g., female gender is the reference category.

*OR* odds ratio, *CI* confidence interval.

## Discussion

The study showed that there are some physical impairments that seem to have particularly high influence on QoL. Particularly noteworthy are pain, contractures, bowel and sexual dysfunction. Some social factors also show high associations with perceived QoL, such as employment, being satisfied with their community health services and having no difficulties in gaining medical aids. The results largely confirm trends seen earlier, but some results also contradict the previous findings. We will focus on the factors that showed the highest effect sizes.

Impairment factors, like lesion height and completeness of lesion, were of lower significance in the association to reported QoL in this survey. A comparison of the literature showed that contradictory statements have already been made in this regard in the past. It has already been reported that, as in our case, there were no relevant association [30], others reported more indirect associations to QoL via physical functioning [31, 32]. Other findings showed that the level of injury in people with SCI had a high impact on their QoL, suggesting that these people need adaptive and compensatory equipment to improve their QoL [33, 34]. Maybe the influence on QoL depends on the technical possibilities for rehabilitation and support in the country of origin of the studies, as these results were reported from Iran and Bangladesh. But also in Canada with high level medical support the injury severity indicated via the physical functioning influence on QoL [32]. Other studys found now significant influence directly to QoL and also referred to indirect effects via physical function and participation [31].

The meaning of the relationship status still seems unclear in relation to other results so far. With *p* = 0.262 there was no significant association to the overall QoL. However, there were contradictory trends on this in earlier reports about the impact on QoL. In a survey of people with SCI for global meaning, the participants named relationship status as one of the five most important factors [35]. Results from Greece showed married life as associated with higher QoL levels (*p* = 0.006) [36]. In Canada it was reported, that being married positively affected life satisfaction [32]. It can be assumed that there are parallels between global meaning, life satisfaction and QoL. However, comparability is limited because the terms describe different nuances in the self-assessment of personal status. In another comparison of 6 different countries, there was no association of the relationship status with QoL [31].

The largest effect size with Cramers’ V (>0,3) showed up for pain, sleep problems, and sexual dysfunction. We also want to go into more detail about some of the other factors that showed high effect sizes in the analysis.

Pain has already shown its high influence on QoL in the literature. One of the studies demonstrated that despite all previous efforts, the presence, complexity, and stability of pain symptoms were refractory to treatment and produced lower QoL ratings in persons with chronic SCI [37]. Several types of pain typically occur in SCI, with central neuropathic pain being a frequent and difficult to manage occurrence [38]. The cyclical relationship of musculoskeletal pain, reduced activity, and maladaptive psychological factors allude to the interdependence of factors, supporting the multidisciplinary approach to care [39]. More precise causes of still high pain levels cannot be deduced from this self-report questionnaire

Sleep problems are a known problem since years. For example in an analysis from Switzerland in 2011 individuals with SCI reported more sleep problems compared to the general Swiss population. This study suggests that clinical screening for sleep issues targeting high-risk groups is needed to reduce the large prevalence of non-treatment in individuals with SCI [40]. The results of the survey do not show us whether the problems are more about falling asleep or staying asleep. However, the causes of sleep disturbances can be various. Psychological problems can cause them just as much as pain, digestive disorders, or sensations in the extremities.

Sexual dysfunction has a high association to QoL and is particularly interesting from a clinical point of view, since it is already known from another analysis of our data that although sexual dysfunction often exist they are nevertheless rarely under medical treatment, or that patients do not seek medical advice for this issue [41]. There may be an issue where QoL can be practically improved through medical assistance.

The logistic regression results are clinically interesting, because they show the probability of experiencing a high QoL in relation to this variables. In addition to the mentioned factors above, there were some more variables worth a consideration.

Bowel dysfunction and diabetes mellitus are challenges for treatment by health professionals. While the influence of bowel dysfunctions is obvious, diabetes is more likely to affect QoL through secondary diseases such as polyneuropathy, or the need for medication such as insulin injections. Unfortunately, multimorbidity is common, with 59.1% of individuals with SCI [32].

The influence of existing contractures on QoL is important to bear in mind. Targeted and multimodal therapy with physiotherapeutic measures, pharmaceutical support and, if necessary surgical release may be required.

Being satisfied with their community health services and having no difficulties in gaining medical aids showed a high odds ratio. This confirms the high value for people with SCI of a good rehabilitation system and generous technical support. The importance has been reported before: technology plays a critical role in promoting well-being, activity, and participation for individuals with SCI. This ranges from lighter wheelchairs to new software, which makes computer interfaces adaptive [42]. Perhaps the importance of the health system and the technical support is also the reason why there are so many differences in QoL between countries. For instance in a study with data from Australia, Brazil, Canada, Israel, South Africa, and the United States of America, analysis of variance showed that living in Brazil was a significant predictor of lower QoL. The differences between the countries could not be explained by differences in demographic and lesion-related characteristics. The results point to the relevance of reintegration of people with SCI into the workforce [31]. Many factors could account for this differences, one could be varying degrees of technical support in the different countries.

As unemployment has been shown to be a risk factor for low QoL, vocational support measures are also an important option and should be implemented early in the rehabilitation strategy. Further discussion on this point has already been published by our study group [43]. However, work can also have a negative influence if it is overstrained. Cross-sectional data from 386 employed men and women with SCI from the Netherlands, Switzerland, Denmark, and Norway were analyzed and work stress and low job control was linked to decreased general QoL [44].

The results regarding age and time since the onset of SCI were largely consistent with previous studies. Declining QoL coincides in the normal population with increasing age. That time elapsed since the onset of SCI mostly means a higher QoL is usually attributed to a higher acceptance and better adaptation to the functional impairments due to acquired SCI [31, 34, 36, 45]. However, there have also been results where QoL did not deteriorate with increasing age. In these studies, it was considered that the positive aspects of getting used to SCI outweighed the general trend of decreasing QoL with age [39].

### Strengths

A major advantage of this analysis is the questionnaire, which asks very broadly about many aspects of daily life. It was developed centrally by the InSCI study group and is thus binding for all InSCI participating countries. This allows a direct comparison between countries of all different WHO regions. This can facilitate the discussion of differences and promote improvements in the context of a “learning health system”.

Another advantage is the largest study population (*n* = 1479) of all participating countries within InSCI.

## Limitations

The recruiting strategy included possible selection bias because all invited persons were treated at least once in one of the specialized SCI clinics. It is possible (and even probable), that the supply situation of persons with SCI who have not been treated in such a center is even worse. The response rate of 32.6% was acceptable but not very high compared to some other surveys. This could be caused by the extensive questionnaire. Another selection bias is possible because answering the questionnaire itself is a challenge for people’s mental ability and motor activity. This may have excluded people who were severely impaired and had no personal assistance to fill in. Regarding the interpretation, it must be taken into account that QoL was assessed only on the basis of one question about the perceived overall situation of QoL and this only according to the last 30 days. Seasonal deviations for example are not recorded with it. In the regression model about 40 % could be explained. This is statistically a good value, but it also shows us that we cannot yet fully explain many associations and, above all, interactions of variables.

The dichotomisation of the variables could possibly lead to a distortion of the results.

## Conclusion

The results of this study provide many indications, but these must certainly be examined in more detail in further studies to determine the cause and options for improvement. However, multifarious factors have been identified that show a high association with the perceived and reported general QoL. From a medical point of view, these are particularly important, as they are modifiable and can lead to practical consequences for possible adjustments of support or medical measures. The importance of a good health care system with adapted rehabilitation and specific support is emphasized by the described complaints and needs. However,the measures must also be adjusted to the financial and infrastructural possibilities of the region in question. It remains important to work closely with each individual person with SCI, since only as a team with doctors, therapists, and technicians, supported by social or political measures, can the goal of providing the best possible care be achieved. Only then can barriers be broken down, impairments reduced, and the Quality of Life noticeably improved.

## Data Availability

The original data are available on request to Mrs. Bökel, Hannover Medical School, Germany or at the InSCI Study Center at the Swiss Paraplegic Center, Nottwil, Switzerland.
